# Statistical test for tolerability of effects of an antifungal biocontrol strain on fungal communities in three arable soils

**DOI:** 10.1111/1751-7915.12595

**Published:** 2017-01-23

**Authors:** Kai Antweiler, Susanne Schreiter, Jens Keilwagen, Petr Baldrian, Siegfried Kropf, Kornelia Smalla, Rita Grosch, Holger Heuer

**Affiliations:** ^1^ Department for Biometry and Medical InformaticsOtto‐von‐Guericke University MagdeburgMagdeburgGermany; ^2^ Department of Epidemiology and Pathogen DiagnosticsJulius Kühn‐Institut – Federal Research Centre for Cultivated PlantsBraunschweigGermany; ^3^ Department of Biosafety in Plant BiotechnologyJulius Kühn‐Institut – Federal Research Centre for Cultivated PlantsQuedlinburgGermany; ^4^ Laboratory of Environmental MicrobiologyInstitute of Microbiology of the CASPragueCzech Republic; ^5^ Leibniz Institute of Vegetable and Ornamental CropsGrossbeerenGermany; ^6^ Present address: Department of AgroEcologyRothamsted ResearchWest Common, Harpenden, HertfordshireAL5 2JQUK

## Abstract

A statistical method was developed to test for equivalence of microbial communities analysed by next‐generation sequencing of amplicons. The test uses Bray–Curtis distances between the microbial community structures and is based on a two‐sample jackknife procedure. This approach was applied to investigate putative effects of the antifungal biocontrol strain RU47 on fungal communities in three arable soils which were analysed by high‐throughput ITS amplicon sequencing. Two contrasting workflows to produce abundance tables of operational taxonomic units from sequence data were applied. For both, the developed test indicated highly significant equivalence of the fungal communities with or without previous exposure to RU47 for all soil types, with reference to fungal community differences in conjunction with field site or cropping history. However, minor effects of RU47 on fungal communities were statistically significant using highly sensitive multivariate tests. Nearly all fungal taxa responding to RU47 increased in relative abundance indicating the absence of ecotoxicological effects. Use of the developed equivalence test is not restricted to evaluate effects on soil microbial communities by inoculants for biocontrol, bioremediation or other purposes, but could also be applied for biosafety assessment of compounds like pesticides, or genetically engineered plants.

## Introduction

The emergence of high‐throughput sequencing techniques now allows a detailed analysis of how microbial communities are influenced by the environmental application of microbial inoculants (Trabelsi and Mhamdi, 2013), pesticides (Jacobsen and Hjelmsø, 2014), transgenic crops (Verbruggen *et al*., 2012) or other human activities with a potential risk for microbial ecosystem services. The effect on the microbial community structure has to be assessed on the basis of high‐dimensional abundance data, typically considering several hundreds or thousands of different operational taxonomic units (OTUs), while the number of samples in typical studies is small. Statistical methods for such high‐dimensional data are available (DeSantis *et al*., 2007; Kropf *et al*., 2007; Kropf and Adolf, 2009; Ding *et al*., 2012) but are usually directed to detect differences between groups representing treatments, soil types, cultivars, etc.. In contrast, statistical methods to show that differences among microbial communities are negligible still have to be established for ecological risk assessment of human activities to support decision making (Heuer *et al*., 2002; Suter, 2006; Weinert *et al*., 2009). This inversed problem is investigated in statistical equivalence tests. In univariate equivalence tests, a tolerance threshold for the dependent (target) variable is defined that is just acceptable as difference for the mean expected outcome of the two groups to be considered as sufficiently similar. Then, modifications of the classical tests for difference are used to show that the real differences are smaller than this limit with probability of at least 1 – α (α is the significance level of the test). That method can be extended to the case of several target variables (low‐dimensional multivariate data). One available method would be the so‐called intersection–union principle, where univariate tests are performed for each dependent variable at the unadjusted alpha level, and multivariate equivalence is accepted if equivalence could be proven in each of the univariate tests. In high‐dimensional data, it would, however, be difficult to define appropriate tolerance thresholds for each variable. Moreover, the claim to prove equivalence in each dependent variable is hard to meet in the high‐dimensional case. Therefore, we utilize multivariate distance measures between the high‐dimensional sample vectors (e.g. Euclidean distances). Chervoneva *et al*. (2007) have carried out this before in a different way. Our approach is more versatile as it allows using non‐Euclidean distances and even dissimilarity measures that do not satisfy the metric axioms of distances. Therefore, we use the term dissimilarity measure in the rest of the article. An ecologically justified limit has to be fixed that defines which distance can be tolerated. The derivation of such an appropriate limit is an essential part of the statistical procedure proposed here.

The application of microbial inoculants is an important component of an environmentally sustainable crop production system. There is an increasing demand for healthy food without chemical residues. In the last years, the market for products based on microbial inoculants, including biofertilizers, plant strengtheners and biocontrol agents, is growing by 10% per year (Berg, 2009). Biocontrol, as part of an integrated pest management, is well suited to partially replace synthetic pesticides which have led to increasing problems with pesticide resistance and which often affect human health and environmental quality (Hajek, 2004; Pérez‐García *et al*., 2011). It is well documented that the treatment of plants with microbial inoculants originated from plant‐associated microenvironments (e.g. soil, rhizosphere, phyllosphere) can efficiently protect plants from pathogens or pests (Berg, 2009; Hallmann *et al*., 2009; Lugtenberg and Kamilova, 2009; Andrews *et al*., 2010; Pérez‐García *et al*., 2011; Kupferschmied *et al*., 2013; Adam *et al*., 2014). However, the application of microbial inoculants to agricultural soils can lead to changes in the indigenous microbial communities, which raises concerns regarding their biosafety (Trabelsi and Mhamdi, 2013). The biocontrol strain *Pseudomonas jessenii* RU47 was isolated from a disease‐suppressive soil and showed antagonistic activity against different phytopathogenic strains of the fungal species *Rhizoctonia solani* and *Fusarium oxysporum* (Adesina *et al*., 2007, 2009). Efficient biocontrol of the important pathogen *R. solani* AG1‐IB was shown in three different soil types, which makes this strain a promising biocontrol agent (Schreiter *et al*., 2014a). Inoculation experiments with strain RU47 gave evidence for at least temporary effects on indigenous bacterial communities in soil (Schreiter *et al*., 2014b). As the control targets of strain RU47 are fungal pathogens, and the observed antibiosis of RU47 against two species of fungi makes non‐target effects likely, it should be evaluated to what extent fungal communities in the agroecosystem are affected. Deep sequencing of barcoded fungal rDNA‐ITS regions amplified from soil DNA (Voříšková *et al*., 2014) provides an excellent opportunity to approach the ecological risk assessment of microbes introduced into agroecosystems to promote cultivated plants. Microorganisms selected for biological control of phytopathogens typically have the potential to produce biocidal compounds like siderophores, antibiotics, biocidal volatiles or lytic enzymes (Saraf *et al*., 2014), which raises concerns in the approval procedure (EC regulation 1107/2009 concerning the placing of plant protection products on the market). However, this physiological potential for non‐target effects might not be ecologically relevant in the environment so that an ecological risk should rather be experimentally assessed *in situ*. Such an experimental approach is impeded by the lack of experimental designs and statistical methods to test for tolerable effects of biocontrol agents on microbial communities.

The objective of this study was the development of a statistical method to test for equivalence of fungal community structures in soil with and without application of a biocontrol agent. This approach was applied to investigate putative effects of the antifungal biocontrol strain RU47 on fungal communities in three arable soils. The effects of RU47 were statistically evaluated with reference to fungal community differences in conjunction with field site or cropping history. Application of such an equivalence test is not restricted to environmental risk assessment of biocontrol agents, but could be widely applied to evaluate effects of strains released for bioremediation or other purposes, of pesticides applied on agricultural fields, or effects of genetically engineered plants on soil microbial communities.

## Results

### Analysis of fungal community structures in three arable soils

**Figure 1:**
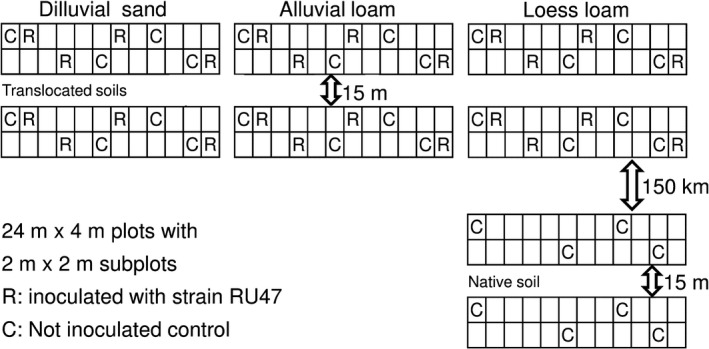
Scheme of the experimental plot systems in Großbeeren (Germany) with three soil types in two blocks, and the field near Klein Wanzleben (Germany), where soils from inoculated and control plots were sampled.

To evaluate the influence of the biocontrol strain *P. jessenii* RU47 on the fungal soil community, bulk soil samples were taken from three soils in separated plots that were treated with strain RU47 in the previous season and from soil untreated with RU47 (experimental station IGZ in Großbeeren, GB), as indicated in Fig. 1. Soil types were diluvial sand (DS), alluvial loam (AL) and loess loam (LL). The three soils have been translocated 40 years ago. Soil LL was also sampled from the original field near Klein Wanzleben (KW; Germany), 150 km apart from GB. The difference in the fungal community structure in soil LL between the two sites reflected an acceptable deviation caused by different weather conditions, crop rotation and agricultural practice. Two blocks in GB were sampled to determine the deviation of the fungal community structure of each soil caused by slightly different cropping histories or random drift due to spatial separation. The differences between these two blocks are considered as alternative approach for defining a threshold for acceptable deviations here.

Fungal ITS regions were amplified and analysed by barcoded high‐throughput pyrosequencing. The amplicon sequencing data were processed by two contrasting strategies to reduce the risk of missing putative effects due to biased assembly of OTUs. The first approach aimed to reliably assign as many sequences as possible to a minimal number of OTUs by a database‐dependent strategy (DBDS). For that, all ITS sequences were assigned to the most similar species hypothesis (SH) in the unite database (Koljalg *et al*., 2013). If a sequence had the same similarity to more than one SH, then it was assigned to the more frequent SH in the dataset. OTUs with low similarity to any fungal ITS were discarded. Thereby, 97.9% of 407 239 sequences were assigned to 1607 OTUs. The 585 OTUs with sequences from at least five samples were further analysed, representing 95.5% of all sequences. The second strategy applied a strict quality control of the sequences and a database‐independent assignment of sequences to OTUs using the pipeline SEED (Větrovský and Baldrian, 2013). In the final SEED dataset, 61.4% of all sequences were retained that were assigned to 2688 OTUs. The 828 OTUs with sequences from at least three samples were further analysed, representing 59.1% of all sequences.

**Figure 2:**
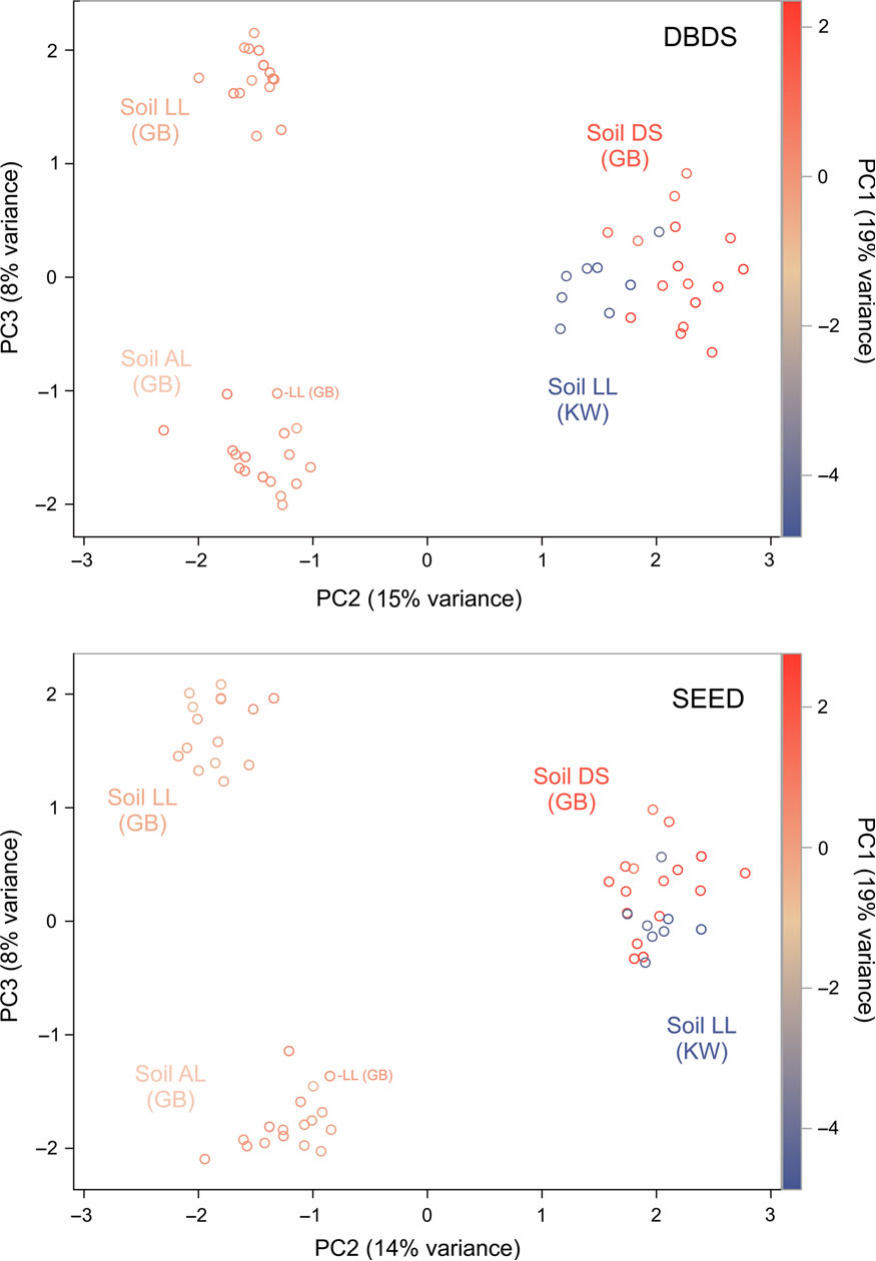
Principal component analysis of fungal communities at two sites, Klein Wanzleben (KW) and Großbeeren (GB), and in three soils (LL for both sites, AL and DS for site GB). Fungal OTU tables were retrieved by two contrasting strategies for sequence assignment, DBDS (upper plot) or SEED (lower plot), as explained in the text. The first principal component (PC1) is indicated by a colour gradient.

**Figure 3:**
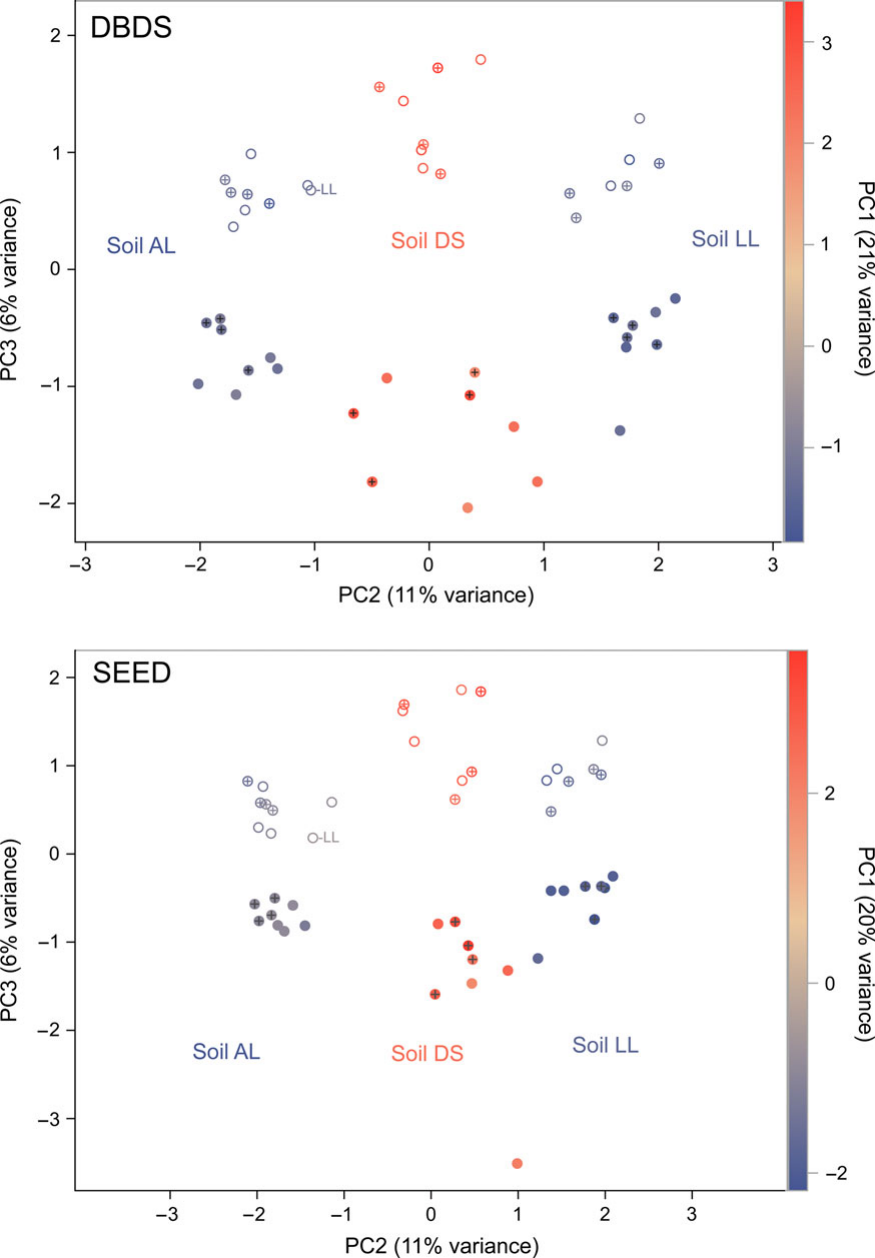
Principal component analysis of fungal communities in three soils sampled at the experimental plot systems in Großbeeren (Fig. 1). Samples from plots treated with the bacterial inoculant strain RU47 are indicated by a cross. Full or open circles indicate samples from adjacent blocks. The first principal component (PC1) is indicated by a colour gradient. The underlying OTU tables were generated by two contrasting strategies for sequence assignment, DBDS (upper plot) or SEED (lower plot), as explained in the text.

**Figure 4:**
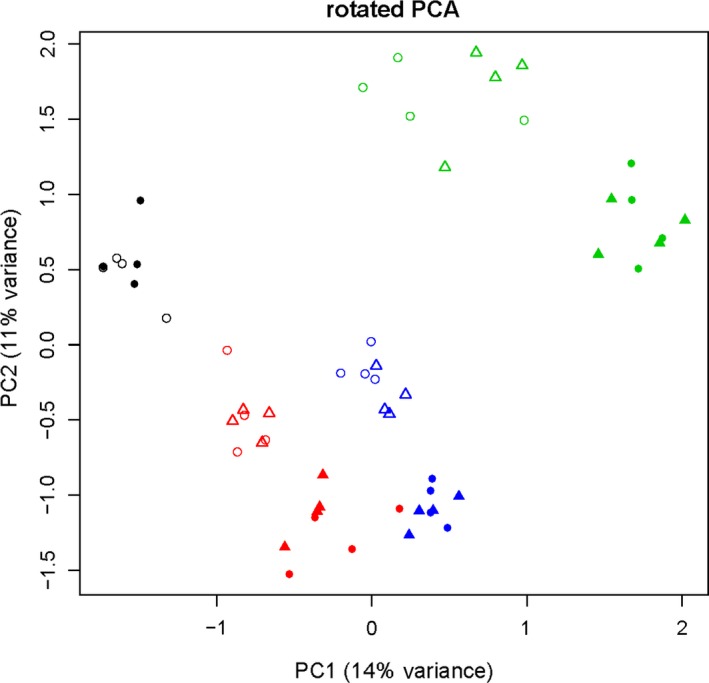
Rotated principal component analysis of fungal communities at two sites, Klein Wanzleben and Großbeeren, and in three soils (LL red–black in Klein Wanzleben, AL blue and DS green). Triangles indicate RU47‐treated plots and circles controls. Full or open symbols indicate samples from adjacent blocks. Fungal OTU tables were retrieved by sequence assignment strategy DBDS, as explained in the text.

The method to generate the OTU‐abundance table, either by DBDS or by SEED, did hardly affect the representation of the fungal community structure (Figs 2 and 3). The fungal communities in the three soils in site GB and in soil LL in sites GB and KW were clearly separated in principal component analysis (Fig. 2). The fungal communities from the loamy soils LL and AL in GB were highly similar on the first and second principal components, but well separated on the third principal component with the exception of a single sample from LL which clustered with the AL samples. Communities of soils DS (GB) and LL (KW) were least similar, as these were best separated on the first principal component which explained more of the variance than the second and third principal component. The first three principal components explained slightly less of the variance for the SEED dataset compared to DBDS. The long‐term spatial separation of the two blocks in GB (15 m apart) is reflected by differences in fungal community structure (Fig. 3, where site KW is omitted from the analysis). The block effect is most evident in the third principal component. The soil type has a much stronger influence on the fungal community structure, as this effect is reflected by the first and second principal components (Fig. 3). The block effect is stronger when phylogenetic information is used as additional source of information. Then, it is visible also with inclusion of soils from both sites even though this spatial effect is not evident for the field site LL (KW) where the soil is mixed by tillage (Fig. 4).

Most of the fungal ITS in all three soils analysed belonged to the *Ascomycota*, which comprised about 72% in the loamy soils AL and LL and considerably less (60%) in the sandy soil DS (Table 1). *Basidiomycota* and *Zygomycota* were also major phyla in these soils with on average 14% or 11% respectively. Their relative abundances significantly differed between soils with specifically low abundance of *Basidiomycota* in soil LL and *Zygomycota* in soil AL. *Chytridiomycota*,* Glomeromycota* and *Rozellomycota* were rather minor components of the fungal communities (Table 1). The most abundant families in all three soils were *Nectriaceae* and *Mortierellaceae*. Their relative abundance significantly differed among soils and was negatively correlated (*R* ^2^ = 0.38). On genus level, fungal ITS assigned to *Cryptococcus* and *Mortierella* were most frequently detected in all three soils, but with significant differences between soils (Table 2). *Mortierella* was especially abundant in the loamy soils AL and LL, while *Cryptococcus* was highest in the sandy soil DS. The abundance of the phytopathogenic species *Rhizoctonia solani*, which was the target of the biocontrol strain RU47, was below detection limit.

**Table 1:** Structure of the fungal communities on phylum level in soils treated with the biocontrol strain RU47 or controls (C) based on the assignment of ITS sequences by DBDS

Phylum	Percentages of sequences assigned to phylum ± SE (*n* = 8)						
	Soil DS (GB)		Soil AL (GB)		Soil LL (GB)		Soil LL (KW)
	C	RU47	C	RU47	C	RU47	C
*Ascomycota*	59 ± 5	65 ± 3	73 ± 2	70 ± 1	75 ± 2	75 ± 1	73 ± 1
*Basidiomycota*	23 ± 5	13 ± 2	17 ± 2	18 ± 2	10 ± 1	8 ± 1	13 ± 2
*Zygomycota*	14 ± 2	16 ± 2	6.5 ± 0.4	7.9 ± 0.6	11 ± 1	12 ± 1	12 ± 1
*Chytridiomycota* a	2.6 ± 0.4	3.4 ± 0.6	2.3 ± 0.3	3.2 ± 0.8	2.4 ± 0.3	3.4 ± 0.6	1.2 ± 0.1
*Glomeromycota*	1.0 ± 0.8	0.1 ± 0.0	0.2 ± 0.1	0.2 ± 0.1	0.7 ± 0.4	0.5 ± 0.3	0.1 ± 0.0
*Rozellomycota* a	0.3 ± 0.1	1.3 ± 0.2	0.1 ± 0.0	0.2 ± 0.0	0.1 ± 0.0	0.3 ± 0.1	0.1 ± 0.0
Unidentified	1.3 ± 0.1	1.9 ± 0.5	1.0 ± 0.5	0.7 ± 0.1	0.8 ± 0.1	1.2 ± 0.3	0.4 ± 0.1

a . Significantly different abundance in RU47‐treated samples compared to controls (univariate stratified permutation tests, unadjusted *P *<* *0.05).

**Table 2:** Relative abundances of the most frequent genera of the fungal communities in the analysed soils based on the assignment of ITS sequences by DBDS

Genus	Percentages of sequences assigned to the genus ± SE (*n* = 8)						
	Soil DS (GB)		Soil AL (GB)		Soil LL (GB)		Soil LL (KW)
	C	RU47	C	RU47	C	RU47	C
*Cryptococcus*	9 ± 1	8 ± 1	15 ± 2	15 ± 2	6.2 ± 0.9	4.8 ± 0.4	3.3 ± 0.9
*Mortierella*	13 ± 2	15 ± 2	6.4 ± 0.4	7.7 ± 0.6	11 ± 1	12 ± 1	12 ± 1
*Pseudeurotium*	0.7 ± 0.3	0.4 ± 0.3	0.4 ± 0.1	0.4 ± 0.1	5 ± 1	5 ± 1	0.9 ± 0.2
*Humicola*	3.9 ± 0.5	4.5 ± 0.4	3.0 ± 0.6	2.4 ± 0.3	2.0 ± 0.4	1.8 ± 0.3	1.3 ± 0.1
*Tetracladium*	3.8 ± 0.5	3.7 ± 0.7	3.3 ± 0.5	3.6 ± 0.5	4.4 ± 0.6	3.8 ± 0.6	0.7 ± 0.1
*Guehomyces*	7 ± 4	1.5 ± 0.5	0.5 ± 0.1	0.4 ± 0.1	2.2 ± 0.7	1.6 ± 0.3	6 ± 1
*Chaetomium*	1.9 ± 0.3	1.7 ± 0.3	1.0 ± 0.1	0.7 ± 0.1	2.5 ± 0.3	3.1 ± 0.4	0.7 ± 0.1
*Cladorrhinum*	1.8 ± 0.6	1.4 ± 0.3	2.8 ± 0.6	2.0 ± 0.4	1.8 ± 0.3	1.6 ± 0.4	0.1 ± 0.0
*Fusarium*	0.6 ± 0.1	0.5 ± 0.1	2.2 ± 0.2	2.2 ± 0.3	2.5 ± 0.5	2.2 ± 0.3	2.5 ± 0.3
*Ascobolus*	0.8 ± 0.4	2.5 ± 2.1	0.0 ± 0.0	0.0 ± 0.0	0.1 ± 0.0	0.1 ± 0.1	0.0 ± 0.0
*Stachybotrys*	0.1 ± 0.0	0.4 ± 0.1	1.1 ± 0.1	1.2 ± 0.1	2.2 ± 0.2	2.2 ± 0.2	0.1 ± 0.0
*Rhizophlyctis* a	0.08 ± 0.03	0.3 ± 0.1	0.04 ± 0.02	0.1 ± 0.0	0.2 ± 0.1	0.2 ± 0.1	0.1 ± 0.0

a . Significantly different abundance in RU47‐treated samples compared to controls (univariate stratified permutation tests, unadjusted *P *<* *0.05). This was also shown for the low abundant genera (< 0.05%) *Entoloma, Ascochyta, Candida, Scytinostroma, Amylocorticium, Pholiota, Serpula, Calonectria, Coccidioides, Hymenoscyphus and Dichotomomyces*.

### Equivalence of fungal communities in soils with and without exposure to RU47

To objectively evaluate whether putative effects of the inoculated biocontrol strain RU47 on non‐target fungi are acceptable in a risk evaluation, a tolerable fungal community change was biologically defined and a statistical test procedure was developed to test whether effects exceed this range or whether the fungal communities are equivalent in this respect. In this study, the tolerable community deviation was defined by two criteria, first the community deviation in the same soil LL between the original field KW and the field plots in GB where the soil was translocated. The second stricter criterion was defined by the deviation between fungal communities in the same soil at the same site GB in two equally treated separated blocks which had slightly different cropping histories.

To statistically prove that an effect is below the threshold at 5% significance level, a one‐sided confidence interval for a meaningful statistic *S* expressing the dissimilarity between treated and untreated soils can be constructed. The interval must lie completely below that threshold. For each soil type, a separate test was carried out as it would not be acceptable to have very similar samples in one soil type overrule dissimilar samples in another type of soil. Given a boundary, the test used OTU counts from soil GB only. Counts from site KW were just used to compute this boundary for the acceptable region that the statistic *S* has to be guaranteed to fall into with a given probability. To calculate that probability a normal distribution‐based approach was used because bootstrap methods did perform too liberal in simulation studies with the given sample sizes. This put a constraint on the choice of dissimilarity measure that can be used in the test procedure as the resulting distribution of the test statistic has to match well enough. The relative Bray–Curtis distance was chosen as dissimilarity measure. The details of the procedure are given in the Experimental procedures section.

Application of the statistical test for equivalence showed with high significance that fungal communities in RU47 treated and in control plots had smaller dissimilarities than the reference thresholds (Table 3). This equivalence was significant for both boundary criteria, the site differences in community structure for untreated LL soils and the stricter block differences. For the latter, the boundary is on average half as high as for the site difference, but still the measured relative Bray–Curtis distance between RU47 treated and control plots is only half of the boundary on average as well. Equivalence of the fungal communities was shown with both datasets, SEED and DBDS.

**Table 3:** Equivalence of fungal communities in RU47‐treated and non‐treated soils

Assessment of upper boundary	Soil	Dataset	Boundary	Bray–Curtis (SD)	Equivalence test (*P*‐value)
Difference among sites (soil LL)	DS	DBDS	0.61	0.24 (0.03)	4.1E‐08
		SEED	0.66	0.26 (0.03)	1.1E‐08
	AL	DBDS	0.61	0.15 (0.02)	3.7E‐12
		SEED	0.66	0.17 (0.02)	1.2E‐12
	LL	DBDS	0.61	0.16 (0.03)	1.1E‐09
		SEED	0.66	0.19 (0.03)	6.0E‐10
Difference among blocks at site GB	DS	DBDS	0.38	0.24 (0.03)	4.8E‐04
		SEED	0.40	0.26 (0.03)	3.9E‐04
	AL	DBDS	0.22	0.15 (0.02)	9.8E‐04
		SEED	0.22	0.17 (0.02)	7.4E‐03
	LL	DBDS	0.25	0.16 (0.03)	4.9E‐03
		SEED	0.26	0.19 (0.03)	1.2E‐02

To validate the method further, we reversed the roles of the block factor and the control/RU47 factor for another analysis. This has not been carried out for biological reasons but to demonstrate the sensitivity of the procedure on the choice of the boundary. Now it is tested if the different blocks of the same kind of soil and the same treatment (control/RU47) can be proven equivalent. The boundary criterion that is constructed from differences among sites remains unchanged. The second boundary criterion now is constructed from the differences between control and RU47 plots. The tests are significant for the loam soils LL und AL when the site differences are used as criterion. All other tests are not significant (Table 4).

**Table 4:** Equivalence of fungal communities in different blocks

Assessment of upper boundary	Soil	Dataset	Boundary	Bray–Curtis (SD)	Equivalence test (*P*‐value)
Difference among sites (soil LL)	DS	DBDS	0.61	0.38 (0.03)	8.9E‐01
		SEED	0.66	0.40 (0.03)	2.6E‐01
	AL	DBDS	0.61	0.22 (0.03)	2.6E‐05
		SEED	0.66	0.22 (0.03)	4.7E‐04
	LL	DBDS	0.61	0.25 (0.03)	7.6E‐04
		SEED	0.66	0.26 (0.03)	1.1E‐03
Difference among control/RU47 at site GB	DS	DBDS	0.24	0.38 (0.03)	1.0E‐00
		SEED	0.26	0.40 (0.03)	1.0E‐00
	AL	DBDS	0.15	0.22 (0.03)	9.9E‐01
		SEED	0.17	0.22 (0.03)	9.3E‐01
	LL	DBDS	0.16	0.25 (0.03)	1.0E‐00
		SEED	0.19	0.26 (0.03)	9.8E‐01

Even though the blocks of each of the loam soils have to be considered equivalent if site differences are considered a negligible difference, they are different enough that RU47 treated soils and their controls have to be considered equivalent in comparison to them.

### Testing for (tolerable) effects of strain RU47 on fungal communities in the three soils

Although the equivalence tests above have shown that the putative effects of RU47 on the fungal communities are tolerable, that does not mean that they do not exist at all. Therefore, we looked with multivariate methods for differences between fungal communities at location GB with and without previous exposure to RU47. Principal component analysis suggested small differences between the fungal composition of inoculated and non‐inoculated soils, albeit much smaller than those between different soils or blocks (Fig. 3).

To test these putative differences for significance, we used two different recently developed tests for high‐dimensional data. The first one (called PCuniRot) uses principal components in a very condensed test statistic (Ding *et al*., 2012). Significance is assessed in repeated computations of the test statistic in rotated samples. The second test version (called Pearson test here, Kropf and Adolf, 2009) uses a multivariate similarity measure, in this case the Pearson correlation coefficients. Both tests have been applied to the DBDS version of the OTU table as well as to the SEED version, always using the log‐transformed abundances and including all three soils in a common analysis. We used factorial models with three factors for the effects of soil type, block and inoculation (Table 5). As can be seen from the *P*‐values of all test versions and from the *R* ^2^ values for the Pearson versions, the soil type was the major influence on the fungal community. The block effects were also highly significant but distinctly smaller in the effect measure *R* ^2^. Inoculation effects of RU47 were by a magnitude smaller in the effect measure. Nevertheless, they were statistically significant in nearly all versions with exception of the SEED version of the PCuniRot test. Interactions between soil type and inoculation were significant only in the DBDS versions with only very small effect measures. The latter fact is also illustrated in Fig. 3, where for soil LL, the dots with crosses (RU47‐treated plots) are shifted a bit compared to the dots without crosses in the third principal component, whereas this effect is not seen so clearly in the other soils.

**Table 5:** Multivariate statistical tests on the effects of RU47, soil type or block on the fungal community structure

	*P*‐value from PCUniRot test		*P*‐value (*R²*) from Pearson test	
Effect	DBDS data	SEED data	DBDS data	SEED data
RU47	0.033	0.120	0.006 (0.014)	0.005 (0.013)
Soil	< 0.001	< 0.001	< 0.001 (0.433)	< 0.001 (0.723)
Block	< 0.001	< 0.001	< 0.001 (0.066)	< 0.001 (0.316)
RU47 × Soil	0.043	0.368	0.008 (0.001)	0.775 (0.002)

To identify putative responders to RU47, multiple univariate stratified permutation tests on effects of RU47 on fungal groups on different taxonomic levels were carried out. The low abundant phyla *Chytridiomycota* and *Rozellomycota* tended to increase in RU47‐treated plots, resulting in unadjusted *P*‐values below 0.05 (Table 1). None among the abundant families and only *Rhizophlyctis* among the abundant genera gave some evidence for a response to RU47 albeit not a decrease (Table 2). Table 6 shows the OTUs with most probable responses to RU47 as revealed by stratified permutation tests. Responding OTUs from SEED were assigned to the genera *Mortierella*,* Cylindrocarpon*,* Cryptococcus*,* Myrothecium* and an unidentified SH of the *Ascomycota*. Responding OTUs from DBDS were assigned to *Cryptococcus terricola*,* Spizellomyces dolichospermus* and an unidentified SH of the *Rozellomycota*. Less abundant responders (< 0.1%) did not contribute much to the result of the multivariate tests. The *P*‐values either decreased or hardly changed when they were removed from the SEED OTU table (0.023 or 0.004 for PCUniRot or Pearson test, respectively), while removing the high abundant responders resulted in higher *P*‐values (0.33 or 0.09 for PCUniRot and Pearson test, respectively), indicating that changes in the relative abundance of these OTUs mostly contributed to the significance of the putative RU47 effects.

**Table 6:** OTUs showing a response to RU47 inoculation as indicated by univariate stratified permutation tests (unadjusted *P *<* *0.05) for fungal OTUs with at least 0.1% abundance

Data	SH	Unadjusted	Genus, Phylum / Species	BLASTN% identity	No. of sequences^a^	
	(07FU)	*P*‐value			C	RU47
SEED	SH183635	0.002	*Mortierella, Ascomycota*	100.0	598	1231
	SH202969	0.038	*Cylindrocarpon, Ascomycota*	100.0	370	498
	SH183335	0.004	*Geomyces, Ascomycota*	98.6	311	346
	SH190017	0.016	*Cryptococcus, Basidiomycota*	100.0	51	279
	SH204317	0.002	Unidentified, *Ascomycota*	99.3	137	89
	SH174294	0.023	*Cylindrocarpon, Ascomycota*	100.0	82	160
	SH175276	0.039	*Myrothecium, Ascomycota*	99.5	78	155
DBDS	SH190017	0.007	*Cryptococcus terricola*	100.0	133	679
	SH183868	0.035	*Spizellomyces dolichospermus*	99.6	27	507
	SH180899	0.038	Unidentified, *Rozellomycota*	96.8	64	177

a Sum of fungal ITS sequences in all three soils (GB) adjusted according to the total numbers of sequences from RU47‐treated and control plots.

## Discussion

In this study, we developed a statistical procedure to test whether an inoculated microbial strain has at most an acceptable effect on the indigenous microbial community. By the application of this test procedure, we showed that the antifungal inoculant RU47 that targeted the disease caused by *R. solani* had such a minor effect on the fungal communities in the three soils that the inoculation can be considered as practically equivalent. The method of how the high‐throughput amplicon sequencing data were processed and assigned to OTUs did hardly affect the results, although two highly contrasting methods were chosen, SEED and DBDS. Probably the most delicate part of the equivalence test is the determination of the thresholds for a tolerable change in the microbial community. We determined that boundary experimentally. This presupposes the existence of an influence of other factors on the sample elements or a proper subset of the samples. The effect of this influence must be acceptable from an ecological point of view. We considered the deviation caused by having the same type of soil in different locations and under different agricultural practice as a starting point, as such influences on fungal communities are generally accepted and have never been associated with any risk. We were able to make this difference easier to accept by not only having the same type of soil but actually the same soil separated years ago in a region of comparable climatic conditions. We choose the difference between non‐inoculated LL soils at these two sites as a boundary for the acceptable region. It might be reasonable to question the acceptability of the difference between the LL soil sites as boundary for the other types of soil. To back up the evidence provided by this boundary, we constructed a second type of boundary as well. The drift in fungal community structure due to separation of soils in two blocks at the site GB and slight differences in cropping history of these blocks provided us with a measurable boundary for each type of soil that should be even more generally acceptable.

Samples that are used to determine the boundaries have to be checked to exclude effects of unusual variation. With increasing number of replicates, this becomes less important. We found one replicate of soil LL in GB that was more similar to soil type AL than to the samples from soil LL in a principle component analysis (Figs 2 and 3). We decided to not exclude this replicate from the analysis since it equally influences the computation of the boundary as well as the difference between control and inoculated groups. As the latter has to be significantly smaller than the former, its effect would in the case of a failure of the first kind increase the distance between the test groups more than it would increase the boundary, thereby decreasing the probability that this failure might occur. All *P*‐values for the test of equivalence stayed significant when that one LL replicate was excluded from boundary determination or from the whole analysis. Special care was taken to not unnecessarily increase the complexity of the computer program that was written to test the data, simulate the experiment to guide the choices in the construction of the algorithm and validate its non‐liberality on bootstrapped samples from the real data.

The relative Bray–Curtis distance was chosen as dissimilarity measure because it was mostly conservative in our studies that used random simulated data and was completely conservative in our simulation studies that used bootstrap samples from the real dataset. Apart from its ecological explanation, the Bray–Curtis distance also has meaning as an information theoretical divergence and has the basic structure of some phylogenetically enhanced distances, as explained in the supplement. While some authors prefer to use model‐based approaches (Warton *et al*., 2012), others show that transformed data analysed with the usual methods work just as well (ter Braak and Šmilauer, 2015). We also believe that there is no theoretical framework that describes ecological data perfect so far, although we prefer to see the samples as empirical distributions of OTUs when they are assessed in its entirety (instead of assessed by their most characteristic OTU).

To estimate the standard deviation, a two‐sample jackknife procedure avoiding systematic underestimation of the real standard deviation under given conditions (Karlin and Rinott, 1982) was used. The estimation of the quantile of the statistic S (that also is used in the calculation of the *P*‐value) is the only step in the procedure that depends on normality. Asymptotically that assumption is met, but sample sizes are usually small in current ecological studies. As it is the statistic S that has to be normal and not the single OTU, it is not straight forward to convince one selves of this property given a small sample set. Because normality is only a sufficient condition for non‐liberality of the confidence interval, we used bootstrapped samples to test the coverage rate of that interval directly. The coverage rate was 100% in 10 000 runs of simulation. We observed the first confidence interval that missed its parameter once when we dropped to 30% confidence. The situation was different in the planning phase, where we used normal distributed OTUs and saw liberal estimations for some sets of low‐dimensional simulation parameters although the Bray–Curtis distance behaved relatively well and never dropped below 90% coverage. Some statistically motivated dissimilarities that we constructed dropped below 20%. We designed the procedure to be applicable to as many meaningful measures as possible, including measures that utilize phylogenetical information (Fukuyama *et al*., 2012).

Different equivalence tests can be combined without the need to adjust the level of the tests. For instance, if there were some OTUs known that should under no circumstances change in relative abundance beyond a known limit, standard univariate equivalence tests could be calculated for each of these OTUs additionally to the community‐based approach described here. Only if each of the tests was significant with unadjusted *P*‐value, equivalence is proven. Each test added to that procedure decreases the power of the procedure. Those OTUs that are checked separately do not need to be included in the community‐based approach. This also holds true for OTUs that are the intended targets of the inoculated strain. The same principle can be used to show equivalence in multiple kinds of populations like bacteria or mesofauna depending on expected effects. The results of the equivalence tests presented in this paper would not change, but equivalence would only be assumed if the tests results for bacteria or other groups were significant, too. We did not consider this when we planned the experiment, because RU47 targets fungi and it is there where we suspect its strongest effects. Although we believe that is true, statistically everybody has the right to doubt it, because significant equivalence tests only prove equivalence (in respect to the boundary values) in those variables that actually are analysed. This trivial fact is much more important in tests for equivalence than it is in tests for difference, where a difference anywhere shows that groups differ. We do not intend to propose focussing on single populations as a standard for good agricultural research practices – especially given the dropping costs for analyses.

Logically related to the question what should be measured is the question when to measure it. The effect of a treatment often is maximal shortly after it is supplied. In terms of risk assessment, the interesting effects are those that last. We chose a time to gather the samples before a new crop is planted which might be affected by a modified fungal community. We believe that this is the most important point in time to know about any effects.

We tried to incorporate phylogenetic information in our analysis. This performed poorly with the methods that try to combine abundance and phylogenetic data directly as was to be expected regarding the low correlation (r=0.02) between the phylogenetic similarity of pairs of OTUs and the correlation of their abundances in our data. We therefore switched to thinking about how to improve just the PCA plot that we already have. In factor analysis, a given PCA result is sometimes rotated to maximize the correlation of its components with the original variables to increase interpretability. There are far too many variables in this dataset for this strategy to be useful. We thought of rotating the PCA result such that it correlates maximally with phylogenetic information. We found out that this can be carried out with an ordinary canonical correlation analysis if advantage of the dual problem of the PCA is taken and the input matrices are reduced in their dimensions first. The result accentuates the block effect to some extend and shows block, soil and site effect in a two‐dimensional plot (Fig. 4). The types of soil now lay ordered as we would have initially expected: the loamy soils are separated from DS, and the KW samples are next to samples from the same type of soil. We failed to produce a picture of equal quality by random rotations.

The RU47 treatment increased the abundance of the members of the *Chytridiomycota* and *Rozellomycota. Chytridiomycota* are typically saprobic fungi with flagellate gametes, degrading refractory materials such as chitin and can also act as mycoparasites (Barr, 1990; Kirk *et al*., 2008). Most of the diversity of the phylum *Rozellomycota* is known only from environmental sequences (Hibbett and Taylor, 2013). In the SEED dataset, *Nectria* was the only genus that responded to RU47 by decreasing abundance in the treated soils. The members of this genus are typically saprotrophs or parasites of trees (Kirk *et al*., 2008). The increase in putative responders indicated that effects of the inoculum on fungal communities are rather due to the added nutrients than caused by the antifungal activity of RU47.

Compared to the DBDS, the SEED‐based data processing resulted in a substantially lower number of sequence reads that passed the quality control steps. However, the representations of the fungal community structure from both datasets were surprisingly similar (Fig. 3). In multivariate statistical tests of RU47 effects on fungal communities, the smaller SEED dataset was less sensitive than DBDS (PCUniRot, Table 5). In both datasets, SH190017 was identified as a responder to RU47 which was based on 330 assigned sequences for SEED and 812 sequences in DBDS. So DBDS might allow for a better sensitivity using a higher percentage of the sequences and assignment to less OTUs, but with a higher risk of false assignments and thus might increase the noise in the dataset.

The disease suppression effects of beneficial microbial inoculants often are based on an antagonistic mode of action which microbes use to establish in competition with other microbes in a natural ecosystem. The majority of commercially available microbial inoculants belong to the genera *Pseudomonas, Bacillus* and *Trichoderma* which are dominant representatives of the natural soil and plant microbiome (Chet, 1987; Berg *et al*., 2005, 2006; Haas and Défago, 2005). Still, the application of microbial inoculants in the environment is an irreversible process and, if applied to plant‐associated microenvironments such as the root zone in sufficient numbers, may perturb indigenous microbial populations and the ecological functions associated therewith (Bankhead *et al*., 2004; Winding *et al*., 2004). To date, only a few cultivation‐independent studies have focused on the effects of beneficial microbial inoculants with disease‐suppressive activity (Scherwinski *et al*., 2007) or commercialized plant stimulants (Chowdhury *et al*., 2013) on indigenous microbes. The inoculant RU47 investigated in this study showed *in vitro* weak antifungal activity against the target pathogen *R. solani* (Adesina *et al*., 2007) and did not produce known antibiotic substances. We found only minor effects on fungal communities in both soils which were tolerable based on our boundary criteria. However, dependent on the properties of the inoculant the non‐target effects can be more severe. Hence, model studies which assess the impact of various biocontrol strains with different properties on non‐target population are needed for environmental risk assessment. Also, such studies should be part of the development process of potential biocontrol strains and will support economically meaningful decisions in the beginning of the product development. Here, we provide the biometrical tools for the data analysis of such an environmental risk assessment of biocontrol strains, which could be analogously applied to other environmental applications like plant growth promoting microbes, biodegraders, genetically modified organisms or ecotoxicological studies.

## Experimental procedures

### Experimental design, sampling and sample processing

Bulk soil samples were taken from experimental plot systems in Großbeeren (Germany, 52.4° N, 13.3° E) and a field near Klein Wanzleben (Germany, 52.1° N, 11.4° E) as indicated in Fig. 1. The plot systems in Großbeeren contained three different soils which have been translocated there in the year 1972 (Ruehlmann, 2013). Two blocks of plots, 15 m apart from each other, were sampled. Each block consisted of three plots, each of which contained a different soil type and was divided into 2 m × 2 m subplots. Soil types were Arenic‐Luvisol (diluvial sand, DS), Gleyic‐Fluvisol (alluvial loam, AL) and Luvic‐Phaeozem (loess loam, LL) (Rühlmann and Ruppel, 2005). To evaluate the influence of the biocontrol strain *P. jessenii* RU47 on the fungal soil community, samples were collected in spring from plots that were treated with strain RU47 in the previous season and from untreated control plots (*n* = 4 per treatment, soil and block). In the preceding season, each lettuce seedling was treated with 2 × 10^8^ cells of RU47 one week before planting and with 3 × 10^9^ cells two days after planting in the field (Schreiter *et al*., 2014b).

On the same day, samples were taken in the field near Klein Wanzleben from where soil LL originated. The difference in the fungal community structure in soil LL between the two sites, Großbeeren and Klein Wanzleben, gives an estimate of acceptable deviation caused by different weather conditions and crop rotations. The two sites are 150 km apart. Deviation of the fungal community structures of each soil between the two blocks in Großbeeren, which were spatially separated for 40 years, gives an estimate of random drift of the fungal community or changes caused by slightly different cropping histories of the two blocks. Crops planted in the years 2000 to 2012 were pumpkin, nasturtium, nasturtium, phacelia, amaranth, wheat, pumpkin, nasturtium, wheat, broccoli, wheat, Teltow turnip, lettuce, lettuce for block 5, and pumpkin, nasturtium, pumpkin, amaranth, wheat, wheat, pumpkin, nasturtium, wheat, wheat, lettuce, lettuce for block 6.

Each subplot was sampled by mixing ten cores (30 cm of top soil; 2 cm core diameter) of bulk soil. DNA was extracted from 0.5 g of soil using the FastDNA SPIN Kit for Soil after two 30 s lysis steps with a FastPrep FP120 bead beating system, and further purified by the GENECLEAN SPIN Kit, as described by the manufacturer (MP Biomedicals, Heidelberg, Germany). The fungal ITS fragments were amplified using the primer pair ITS1F (CTTGGTCATTTAGAGGAAGTAA) / ITS4 (TCCTCCGCTTATTGATATGC) as previously described (Weinert *et al*., 2009). The products were purified with a Minelute PCR purification kit (Qiagen, Hilden, Germany). Barcoded amplicon pyrosequencing was performed at the Biotechnology Innovation Center (BIOCANT, Cantanhede, Portugal) on a 454 Genome Sequencer FLX platform according to standard 454 protocols (Roche – 454 Life Sciences, Branford, CT, USA). Briefly, the purified PCR products were used as target to amplify the ITS1 region with fusion primers containing the Roche‐454 A and B Titanium sequencing adapters, an eight‐base barcode sequence in adaptor A, and specific sequences ITS1F / ITS2 (GCTGCGTTCTTCATCGATGC) targeting fungal ribosomal genes. The data were submitted to NCBI SRA with accession number SRP073893.

### Generation of OTU‐abundance tables from ITS sequences

The amplicon sequencing data were processed by two contrasting strategies. DBDS aimed to reliably assign as many sequences as possible to a minimal number of OTUs. For that, all ITS sequences were assigned to the most similar species hypothesis (SH) in the UNITE version 7.0 database (Koljalg *et al*., 2013) using Megablast (Camacho *et al*., 2009). The SH database is available at https://unite.ut.ee/repository.php. If a sequence had the same bit score to more than one SH, then it was assigned to the most abundant SH in the dataset. For processing the megablast results, the java tool blastparser was written and integrated into a Galaxy workflow (https://galaxyproject.org). It makes a unique assignment of the sequences to an OTU and generates the OTU‐abundance table. OTUs were discarded when the assigned sequences were < 95% similar to all SH, or had < 100‐bp alignment length or had highest similarity to non‐fungal ITS. The other approach applied the pipeline seed 1.2.1 (Větrovský and Baldrian, 2013) to achieve a strict quality control of the sequences and a database‐independent assignment of sequences to an OTU. Briefly, pyrosequencing noise reduction was performed using the denoiser 0.851 (Reeder and Knight, 2010). Chimeric sequences were detected using usearch 7.0.1090 (Edgar, 2010) and deleted. Only sequences longer than 310 bases were retained, and full ITS2 regions of these sequences were extracted using itsx (Bengtsson‐Palme *et al*., 2013). Full ITS2 regions were clustered using UPARSE implemented within usearch (Edgar, 2013) at a 97% similarity level. Consensus sequences were constructed for each OTU, and the closest hits at a genus or species level were identified using blastn against unite version 7 and GenBank for fungi. Ecology was assigned based on genus‐level best hits to those taxa whose genera show consistency in this respect using published data (Tedersoo *et al*., 2014).

### Statistical test for equivalence of fungal communities

The equivalence testing procedure is based on the relative Bray–Curtis distance between fungal communities of the control group and the RU47‐treated group. The procedure computes a test statistic S which represents the dissimilarity between two groups of samples. For that statistic, a conservative approximation of its variance is constructed (Karlin and Rinott, 1982). Our samples consist of relative counts of OTUs which sum to one for each sample, i.e. discrete empirical probability densities on the space of all OTU types. We start by separating our samples corresponding to their stratum and group. We have two groups: treatment and control. In our case, we also have two strata: samples from the first block and samples from the second block. This should not be confused with the distinction of the different soil types, which was considered by completely separate analyses for each soil. All strata that are used in that procedure must be allowed to compensate each other's results. The assumption is that if equivalence is true for one stratum, it also is true for the others. Although it is hard to find a realistic case where variances differ between strata while that assumption is met, the variance estimator is guaranteed to be unbiased or conservative for any statistic, always, but it tends to be suboptimal in terms of power. For each stratum, two discrete empirical probability densities are computed by averaging all its samples that belong to the treatment group for the first distribution and all that belong to the control group for the second distribution. From both empirical distributions, we calculated the relative Bray–Curtis distance as dissimilarity measure, separately for all strata. This can be carried out by calculating the sum of absolute values of the group difference and scaling the result by 0.5, as shown in the supplements. The average of these values over all strata is our test statistic *S*. We used an unweighted average. Instead of averaging the samples, we could have averaged pairwise Bray–Curtis distances. Both are valid procedures, but the approach taken here has the advantage of mitigating the difference between group distance and average pairwise sample distance.

To estimate the variance of S, a stratified two‐sample jackknife procedure was used. Technically, a variance for the test statistic with a reduced number of samples is estimated. At the start of the procedure, a value r is fixed that describes the proportion of sample elements used in each step of the jackknife procedure. For each group j∈{1,2} in each stratum $i\in \left\{1,\ldots ,m\right\},$ a reduced sample size $q_{ij}\;=\dot{r}\cdot n_{ij}$ is calculated where $n_{ij}$ are the original sample sizes. We chose r=3/4, which in our case means that in each combination of stratum and group 3 of the 4 available sample elements are used in a jackknife step. The procedure is repeated over all possible combinations of samples with exactly $q_{ij}$ samples in stratum i and group j (yielding $N\;=\;\prod_{i,j}\left(\begin{array}{c} n_{ij}\\ q_{ij} \end{array}\right)$ runs). In each run, a test statistic $S_{k}^{*}\left(k\;=\;1,\ldots ,N\right)$ is calculated as described above, starting with averaging of the selected samples in each stratum‐group‐entity and ending with averaging the relative Bray–Curtis distances over the strata. No sample is allowed to switch group or stratum. These *N* values of the jackknife test statistics are averaged $\bar{S^{*}}=N^{-1}\sum_{k=1}^{N}S_{k}^{*},$ and the square of the difference between each value and that average is calculated and averaged again and scaled by the fraction of left out samples per entity. The final variance estimate is given by the formula: $\hat{\sigma^{2}}\left(S\right)={\left(1-r\right)}^{-1}N^{-1}\sum_{k=1}^{N}{\left(S_{k}^{*}-\bar{S^{*}}\right)}^{2}$

The value of the test statistic and its standard deviation can be used under assumption of normality to compute the upper limit *UL* of the one‐sided confidence interval for *S* by $=S+t_{1-\alpha ,n-2m}\cdot \sqrt{\hat{\sigma^{2}}\left(S\right)}$ . We used a *t*‐distribution instead of a normal distribution to be on the conservative side. For degrees of freedom, we chose to use n−2m, i.e. the total sample size minus number of groups times number of strata.

The boundary B is calculated in the same way as the test statistic S. In our case where we chose the block factor to compute the boundary, this was carried out by switching the variable group with strata. In the other case where we used the location (GB vs. KWL) for untreated samples of LL soil instead, the location was used in place of group in the procedure described above. Concerning the equivalence test, this also is the only place where samples from the second location where used. There is no need to estimate the variance of B. If S changes systematically with changing sample sizes, B must have the same structure of sample sizes as S has (or the resulting bias should be proved to be conservative, i.e., decrease B in respect to S).

The equivalence test can be finalized by comparing the upper limit *UL* of the one‐sided 95% confidence interval of the test statistic *S* with the boundary *B*. The test is significant (i.e. equivalence is proven) if ≤B. The probability of the set of points right to the equivalence boundary B is the *P*‐value of the test (using the *t*‐distribution as described above).

We suggest using a balanced design, but the implemented program does work for all designs for which it is possible to define r as long as there is no empty resampled entity and the maximal sample size in an entity is below 64. Both latter limitations were introduced for computational convenience and are unrelated to the algorithm described. The first limitation is both for computational and theoretical reasons. This jackknife procedure is only guaranteed to be non‐liberal if the ratio r is constant over all group‐strata combinations. A small deviation from that ratio may be not too far away from the theoretical results. The program accepts an input parameter for a tolerance value to check if the ratio of the number of chosen samples and the sample size is approximately equal to r (e.g. if we would have excluded the one LL sample that clustered with AL soil from the analysis, there would be only 3 samples instead of 4). When three‐fourth of those three samples, i.e. 2.25, are to be selected, only 2 would be selected and checked that | $|{}^{2}{/}_{3}$ – ${}^{3}{/}_{4}|$ | < tolerance value.

The program code including a description (‘README’) and the raw data of this study can be downloaded from ‘https://www.researchgate.net/publication/301770482_data_Statistical_test_for_tolerability_of_effects’, doi: 10.13140/RG.2.1.3287.6407.

### Statistical testing for effects of RU47 on fungal communities

Assignments of sequences to OTUs by DBDS and SEED resulted in two tables representing the OTU‐abundance structure of the fungal communities of all samples. Relative abundances within each sample were log‐transformed (log[relative abundance * 1000 + 1]) to ameliorate deviations from normal distribution. Samples from plot systems in Großbeeren were analysed by multivariate statistics to test for significant effects of the inoculated strain RU47 on fungal communities, while taking the additional factors soil and block into account. Two factorial multivariate statistical tests with three factors were applied. PCUniRot is based on principal component analysis combined with a modified ANOVA test statistic for the framework of a general linear model (Ding *et al*., 2012). This statistic uses a weighted combination of the sums of squares for the first *q* principal components (*q* determined by the Kaiser criterion). Rotation tests are then applied to derive the *P*‐value. The other test (called Pearson test here) is based on Pearson correlation coefficients used as multivariate similarity measures for pairs of sample vectors (Kropf and Adolf, 2009). The test statistic describes the multiple correlations between the similarity measures for all pairs of sample vectors and the corresponding differences in the factor level of the factor of interest for the same pairs of sample vectors after eliminating the influence of all other factors. The *P*‐value for the test is again derived in rotation tests. Additionally, the squared multiple correlation coefficient *R* ^2^ as effect measure can be interpreted as the proportion of variability in the observed similarity measures explained by the factor tested. These tests are more powerful in high‐dimensional settings with small sample sizes than competing tests in many situations (Ding *et al*., 2012).

Individual OTUs which were likely influenced by the inoculation of RU47 were determined by a stratified permutation test (Good, 2000). In contrast to an ANOVA, the concept of this type of test remains valid even for groups with zero variance as was common in our data. The data were not log‐transformed as this test procedure works correct on relative counts. As a test statistic, the sums of the absolute differences between replicates in each group and stratum were added. Stratification allows pooling evidence. Soil type and block were chosen as strata. Control/RU47 labels were randomly permutated 10 000 times. Permutations were restricted to stay inside the same stratum.

### PCA rotation by canonical correlation to phylogenetic similarities

The phylogenetic similarities between all pairs of OTUs are given as a p×p matrix M. The abundance table X in this case is a p×n matrix of relative OTU abundancies transformed using the logarithm and centralized for each OTU afterwards. The motivation of the method suggests double‐centred data, but this would be limited to PCAs on covariance matrices. The way described here also works on top of correlation matrices. We use a covariance matrix, but the results between double‐centred and centring OTUs only were hardly visible. The n abundancy and p similarity columns are different kinds of measurements for each OTU. A canonical correlation between both matrices produces an orthonormal matrix as a result, which can be used as generalized rotation transformation. First, we have to reduce the dimensions of matrices to get meaningful results. The reduced abundance matrix used corresponds to the principle components that were used in our plots. The PCA was performed with the OTUs seen as variables resulting in a n×r matrix Y of principle components. This matrix cannot be used directly in our canonical correlation, because there the OTUs are seen mainly as sample elements which correspond to a transposed view of the problem. But for each PCA exists a dual PCA formulation that we can use, such that $\left(p-1\right)Y=X^{\prime }U\Lambda^{-1/2}$, where the eigenvector column matrix U and eigenvalue diagonal matrix Λ are solutions to the eigenvalue equation $XX^{\prime }U=U\Lambda$. The scaling constant (p−1) and the column‐scaling matrix $\Lambda^{-1/2}$ are not important for our current purposes, because we can standardize the components afterwards as we like. The matrix U, we will use as input for the canonical correlations, and the resulting r×r orthonormal matrix R will be used to define the ‘rotated’ PCAs $\tilde{Y}:=X^{\prime }UR$. The dimension of the phylogenetic matrix M has to be reduced, because it is so big that the columns of U already lie in a subspace of the column space of M, without any rotation. We use a PCA on M as well (i.e. an eigenvalue equation on $M^{\prime }M$) to reduce its dimension and take the k first eigenvectors as input for the canonical correlations. We used Scree‐plots to determine r and k.

Rotating the eigenvectors rearranges the variance that corresponds to each eigenvector (i.e. their eigenvalue). It can be necessary to change the order of those vectors after the procedure described above to choose the vectors that contain the maximum of variance. Because the columns of $X^{\prime }$ are centralized, and those of $X^{\prime }U$ orthogonal (as those of $X^{\prime }U\Lambda^{-1/2}$ are), the columns of $X^{\prime }U$ are uncorrelated. $X^{\prime }UR$ is a linear transformation of those columns; therefore, the variances of its columns can be calculated by adding the variances of the columns $X^{\prime }U$ times the squares of the corresponding factors which are the squares of the entries of R. If λ is the vector of column variances corresponding to Y (i.e. the diagonal entries of Λ ) and $R^{2}$ is the matrix R squared elementwise, the rotated variances are equal to $\lambda^{\prime }R^{2}$. The proportion of total variance can be calculated by dividing that vector by the total variance.

## Conflict of interest

The authors have no conflict of interest to declare.
